# The Identification of First COVID-19 Cluster in Indonesia

**DOI:** 10.4269/ajtmh.20-0554

**Published:** 2020-10-12

**Authors:** Vivi Setiawaty, Herman Kosasih, Yan Mardian, Emita Ajis, Endang Burni Prasetyowati, Siswanto, Muhammad Karyana, SARS-CoV-2 Reference Laboratory, Ministry of Health, Indonesia

**Affiliations:** 1 National Institute of Health Research and Development (NIHRD), Ministry of Health, Jakarta, Indonesia;; 2 Indonesia Research Partnership on Infectious Disease (INA-RESPOND), Jakarta, Indonesia;; 3 Directorate of Surveillance and Health Quarantine, Ministry of Health, Jakarta, Indonesia

## Abstract

We describe the first 11 detected COVID-19 cases in Indonesia, resulting from a local transmission occurring in a club and a restaurant. The virus was detected until an average of 21.3 days (range: 11–25, SD: 4.1) after the onset of illness, and the partial *N* gene sequences (28,321–28,707 nucleotide position) had 100% similarity with the SARS-CoV-2 sequence from Wuhan. Two subjects were asymptomatic, and one subject has died.

Fifteen years after the Avian influenza epidemic,^1^ Indonesia was recently hit by another respiratory outbreak, COVID-19, caused by the novel SARS-CoV-2. This virus belongs to the Coronaviridae family, genus *Betacoronavirus*, together with SARS-CoV and Middle East Respiratory Syndrome (MERS)-CoV that were never identified in Indonesia despite worldwide spread in 2003 and 2012.^2,3^

SARS-CoV-2 was first reported on December 31, 2020 in Wuhan, China, and then it rapidly spread globally to infect more than 3 million people in more than 200 countries by April 30, 2020.^4^ The government has taken several precautions to anticipate the introduction of this virus to Indonesia, such as evacuating students from Wuhan and quarantining them in Natuna Island for 14 days, canceling direct flights from China to Indonesia, observing Indonesians who worked at the Diamond Princess cruise ship in Yokohama, Japan, monitoring the temperature of people coming to Indonesia at the ports and airports, and testing suspected subjects at the NIH Research and Development, Ministry of Health. Recently, PCR can be conducted in 32 laboratories throughout the archipelago, and more than 20,000 people have been infected by SARS-CoV-2 by the end of May 2020.

Two months after the report from China, on March 1, 2020, nasopharyngeal, throat swabs and sputum from 31- and 64-year-old women (case #1 and case #2) were confirmed positive for SARS-CoV-2 by real time (RT)-PCR using two WHO-recommended methods.^5,6^ The Charité Virology, Berlin method uses a two-step assay. The first step is to detect the envelope (E) gene of subgenus Sarbecovirus, and the second step is to detect RdRp gene which is specific for SARS-CoV-2. The U.S. CDC method targets specific SARS-CoV-2 genes (N1 and N2 assays). The first swab specimens from all 11 cases were tested using Berlin and U.S. CDC methods. All showed concordant results (cycle threshold-value < 40). Follow-up specimens were tested only using U.S. CDC primer sets. RT-PCR and sequencing protocol from the Department of Medical Sciences, Ministry of Public Health, Thailand, targeted 397 base pairs of the SARS-CoV-2 *N* gene^7^ for two samples.

Case #1 (31 F) fell ill 2 days after she attended a dancing event, and visited an outpatient clinic on day 4 of illness when she had a persistent fever and cough. She received symptomatic medication (antipyretic and cough syrup). On day 10 of illness, she had breathing difficulty and was hospitalized with bronchopneumonia. Her leukocyte, lymphocyte, and platelet counts were normal (6,100/mm^3^, 36%, 227,000/mm^3^, respectively). Two days later, she informed her pulmonologist that she had contact with a foreigner, who had just been confirmed having COVID-19, while dancing. This foreigner is a Japanese national who lived in Malaysia and arrived in Indonesia just 1 day before the dancing event. She had visited Japan in January 2020 and no other countries apart from that. Case #2 (64 F) met case #1 2 days after the dancing event and had a fever and cough 4 days later. She was diagnosed as having typhoid fever (*Salmonella typhi* Abs titer of 1/120) and hospitalized on the same day. An investigation was immediately conducted, tracing places that she (case #1) and the foreigner had visited, and identifying people with whom they had contact.

Specimens from 33 close contacts of the foreigner (drivers, guide, waiters at a restaurant, and other guests at the dancing event) and 80 close contacts of the first in-country Indonesia case (family members, friends, medical staff) were tested. Eleven contacts were positive, in which nine contacts were ill, and two contacts were asymptomatic. Fever and respiratory symptoms appeared 3.2 (2–7, SD: 1.5) days after exposure. Fever, cough, sore throat, and shortness of breath were reported in seven (78%), seven (78%), four (44%), and three (33%) of the symptomatic patients, respectively. No gastrointestinal, neurological, or dermatological symptoms nor sequelae were reported. All patients only received multivitamins and symptomatic treatment such as antipyretics and mucolytics. Antibiotics were given to some patients. One symptomatic case died on arrival at the hospital 15 days after illness. Among the 11 cases, 10 cases had contact with the foreigner, and one case was a secondary contact. The transmission pattern, incubation period, duration of illness, serologic conversion, and hospitalization days are shown in Figure 1. The proportion of positivity among contacts with the foreigner was 30.3% (10/33) and within friends and family of case #1 was 1.3% (1/80). This finding suggested that transmission is more acute during physical exercise in crowded indoor or enclosed environments,^8^ as has been reported in South Korea.^9^ No positive cases were found among 71 medical staff at the first hospital before case #1 was referred to the reference hospital.

**Figure 1.**
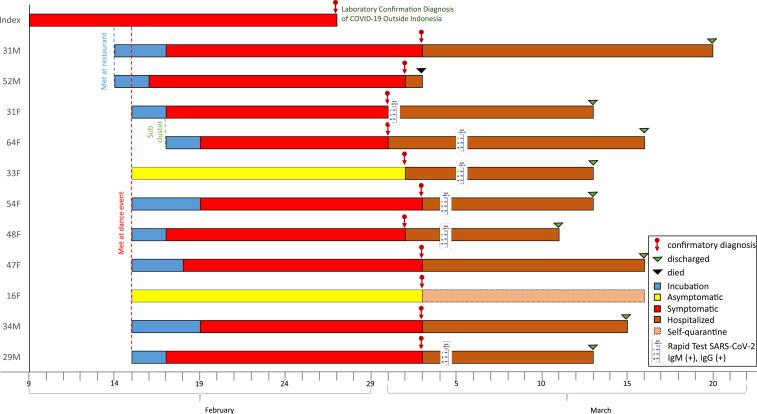
SARS-CoV-2 transmission pattern, incubation period, serologic conversion, and hospitalization days of first Indonesia’s cluster.

Nasopharyngeal and throat swabs from all the positive cases were collected every 1–2 days and tested by RT-PCR. Viral RNA decreased over the course of illness and disappeared on an average of 21.3 days (range: 11–25, SD: 4.1) after symptom onset (Figure 2). Patients were considered cured when no viral RNA was detected in two consecutive day specimens.^10^ SARS-CoV-2 IgM and IgG antibodies were tested in six patients using rapid IgM/IgG duo SD Biosensor kit (SD Biosensor, Gyeonggi-Do, Korea). Sera from four subjects collected on days 13, 14, and 16 of illness were positive for SARS-CoV-2 IgM and IgG. One symptomatic subject had negative SARS-CoV-2 IgM and IgG on day 11, but seroconverted on day 15 of illness, whereas one asymptomatic subject was positive for SARS-CoV-2 IgM and IgG on day 19 after first exposure (Figure 2).

**Figure 2.**
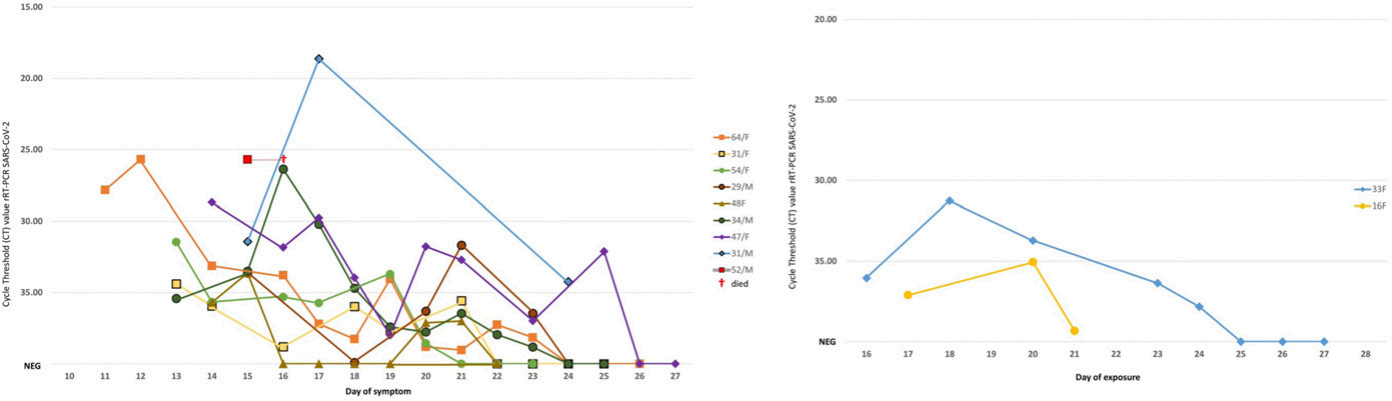
Molecular characterization of symptomatic (left) and asymptomatic (right) cases. The kinetics of virus in serial respiratory specimens (ct value, *Y*-axis). Symptomatic cases (nine patients) were displayed based on the day of symptoms, whereas asymptomatic cases (two patients) were displayed based on the day of exposure.

Direct sequencing from the amplified product of partial *N* gene (387 bp) of SARS-CoV-2 from the case #1 specimen and the case #2 specimen shows 100% similarity with the sequence from the early detected virus in Wuhan.^11^ Based on recent Global Initiative on Sharing All Influenza Data (GISAID) database (S, V, G, and other clade), the two samples were clustered on others/L clade/type (Figure 3).

**Figure 3.**
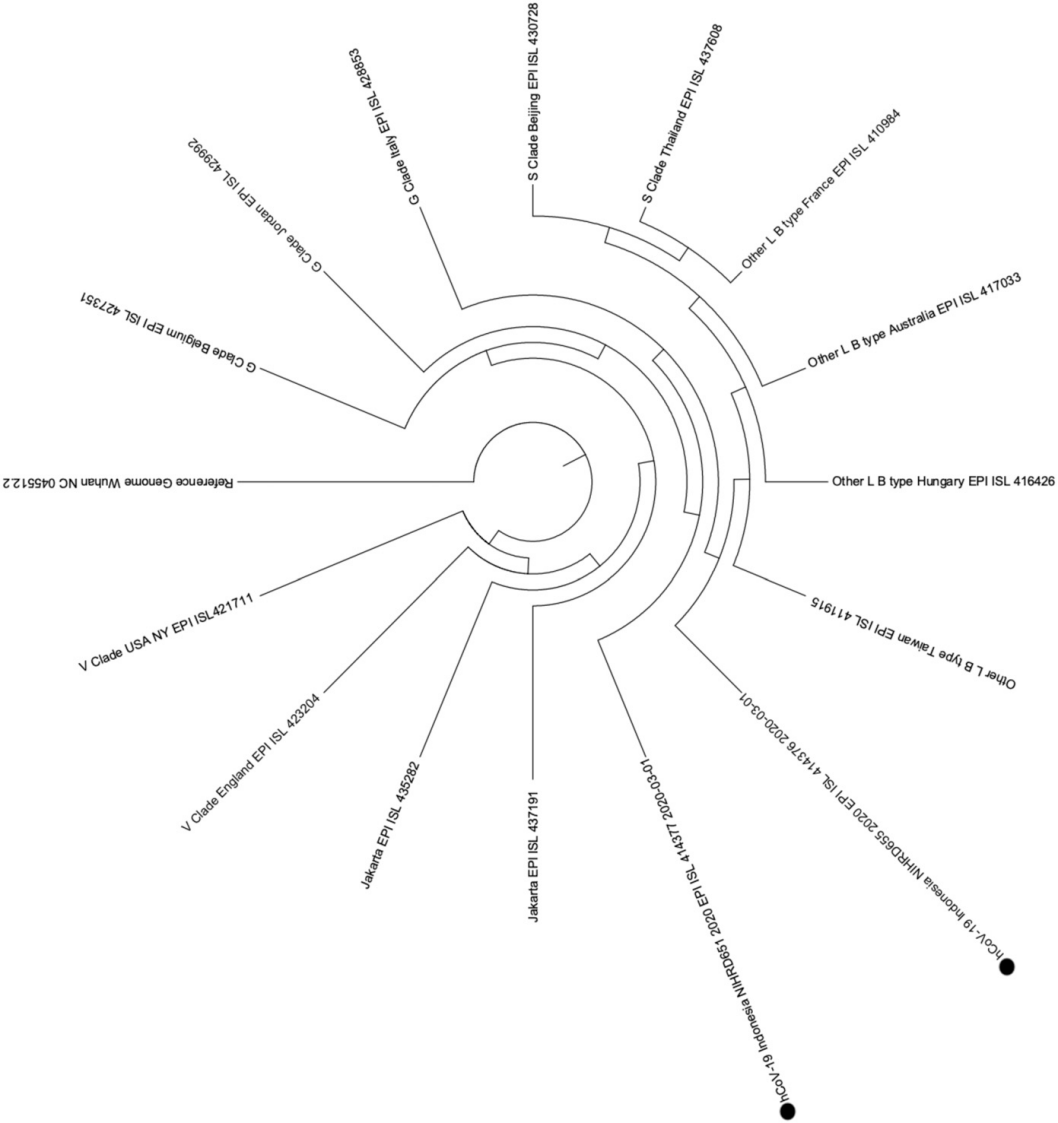
Neighbor-joining phylogenetic tree for two samples from the first cluster in Indonesia (indicated with solid circles). The phylogenetic analysis was performed based on 387 bp of the partial *N* region. The nucleotide sequences obtained from direct sequencing and the reference sequences retrieved from the GISAID database were aligned with Clustal X software (http://clustal.org/clustal2/). The evolutionary distances were computed using the maximum composite likelihood method. The analyses were performed using Molecular Evolutionary Genetics Analysis software version 7 (https://www.megasoftware.net/).

We briefly described the epidemiology, clinical, and virological findings of Indonesia’s first 11 cases occurring in Jakarta, transmitted by a foreigner who had been infected abroad. As the cases were identified 2 weeks after exposure, careful close contact investigation had been conducted and was able to identify more cases. Further monitoring, testing, and contact investigation should be performed to halt the transmission in Indonesia. As the number of cases keeps increasing, Indonesia should take robust measures to contain the spread of this virus, including social distancing, self-isolation for people with signs and symptoms of respiratory infection, increased access to testing, isolation of cases, and intensive contact investigation.
